# Collagen-induced restructuring of astrocytes and vessels in organotypic mouse brain slices

**DOI:** 10.3389/fragi.2026.1910447

**Published:** 2026-08-20

**Authors:** Eva Steiner, Valentin Wolfgang, Christian Humpel

**Affiliations:** Department of Psychiatry and Psychotherapy, Laboratory of Psychiatry and Exp. Alzheimer’s Research, Medical University of Innsbruck, Innsbruck, Austria

**Keywords:** astrocytes, brain vessels, collagen, live cell imaging, reorganization

## Abstract

**Introduction:**

The brain exhibits high plasticity and responds to many forms of external stimuli, such as trauma, seizures, drugs, and biomaterials. Collagen is a non-toxic biocompatible protein that participates in the plastic reformation of the brain. In this study, we explored whether collagen can reconstruct astrocytes and brain vessels by using microcontact printing on organotypic mouse brain slices.

**Methods:**

Organotypic brain slices from postnatal days 8–10 were prepared and connected to collagen microcontact prints. After 2–6 weeks, glial fibrillary acidic protein–positive (GFAP^+^) astrocytes and laminin^+^ vessels were immunohistochemically stained.

**Results:**

Our data show that (1) collagen restructures laminin^+^ vessels in the whole brain slice along 50-µm-wide collagen lanes. (2) This restructuring is linked to the reformation of GFAP^+^ astrocytes in the collagen–laminin lanes. (3) In contrast to 50 µm lanes, larger 400-µm collagen spots caused a marked depletion forming large holes in the brain slice accompanied with the cell death of GFAP^+^ astroglia. (4) Preliminary/exploratory data using live cell imaging may indicate that astroglia restructure before the vessels.

**Discussion:**

Collagen has the distinctive potential to induce plasticity and the restructuring of vessels and astrocytes, however, collagen may also lead to cell death under certain conditions.

## 1 Introduction

The extracellular matrix (ECM) provides biophysical stability, regulates intercellular signaling, and plays a central role in preserving the balance between glial, neuronal, and vascular components. Collagen is the major structural element of ECM (Ricard-Blum, 2011; Ucar and Humpel, 2018; Wareham et al., 2024). It is composed of multiple polypeptide chains and is defined by its hierarchical self-assembly and characteristic triple-helical structure, which ensure biochemical and mechanical stability. In addition to its structural role, collagen mediates critical interactions between the ECM and resident brain cells, a process that is considered essential for repairing central nervous system injuries and diseases (Nimni, 2018; Wareham et al., 2024). Owing to its biocompatibility, nontoxicity, and biodegradability, collagen has attracted considerable attention as a biomaterial for therapeutic applications. It is particularly suitable for studying neuroprotection, neurodegeneration, and brain repair processes (Ucar and Humpel, 2018). In fact, it is interesting to explore how collagen modulates the plasticity of brain cells.

Astrocytes are specialized glial brain cells that, together with neurons, form integral components of the central nervous system and contribute to the stability of neuron–glia networks (Hatten et al., 1991; Verkhratsky et al., 2015; Liu et al., 2017). Glial fibrillary acidic protein (GFAP) constitutes a major part of the intermediate filament network in astrocytes and serves as a reliable marker of astrocyte development and physiological state (Brenner and Messing, 2021). In response to injury, astrocytes undergo reactive gliosis, which is associated with a marked upregulation of GFAP expression (Hol and Pekny, 2015). Astrocytes also play a crucial role in the formation of the blood–brain barrier (BBB) and are structurally connected to blood vessels through specialized processes (Brightman, 1991). Furthermore, astrocytes contribute to the regulation of perivascular homeostasis, nutrient transfer, and vessel maintenance (Cohen-Salmon et al., 2021). The functional and structural integrity of cerebral blood vessels is essential for normal brain function because they constitute the basis of the BBB. The disruption of this barrier is a hallmark of many neurodegenerative disorders and leads to the loss of vascular integrity (Sweeney et al., 2018). Laminin is a glycoprotein that plays a central role in regulating BBB function under both physiological and pathological conditions (Halder et al., 2023; Nasrollahi and Yao, 2025); however, neuroinflammatory processes can impair BBB structure and function (Zapata-Acevedo et al., 2022).

Organotypic brain slices are 3D brain sections that contain all the brain cells in a complex 3D network and can be cultured for several weeks. We have extensive experience in culturing organotypic brain slices in our lab and have studied neurons, astrocytes, and vessels (Humpel, 2015; 2018). Recent evidence shows that microcontact printing (µCP), a novel technique developed in our lab, allows for the selective application of any molecule of interest on brain slices (Steiner and Humpel, 2023). Collagen is a key molecule in µCP for loading these substances onto brain slices (Steiner and Humpel, 2021). By using this method, we also found novel biomarkers for Alzheimer’s disease (AD) on the basis of the migration of neurites (Yilmaz et al., 2024), microglia (Yilmaz et al., 2024), or endothelial cells (Yilmaz et al., 2025) outside and along µCP collagen lanes. In a previous study, we demonstrated that astrocytes are capable of spontaneous reorganization within brain slices along collagen lanes (Steiner and Humpel, 2021). Very recently, we reported that collagen-mediated µCP allows us to perform the live cell imaging of GFAP^+^ astrocytes or laminin^+^ vessels in brain slices (Humpel, 2025).

In this study, we aimed to reproduce our previous finding that astrocytes reorganize on collagen lanes in brain slices (Steiner and Humpel, 2021) and to evaluate this process in more detail. We are particularly interested in demonstrating the interaction between GFAP^+^ astrocytes and laminin^+^ vessels along such collagen lanes over time. We will show that astrocytes and blood vessels undergo pronounced reorganization into a distinct pattern along collagen lanes (50-µm width) within 2 weeks, whereas larger collagen spots (400-µm diameter) induce cell death.

## 2 Methods

### 2.1 Organotypic brain slices

**FIGURE 1:**
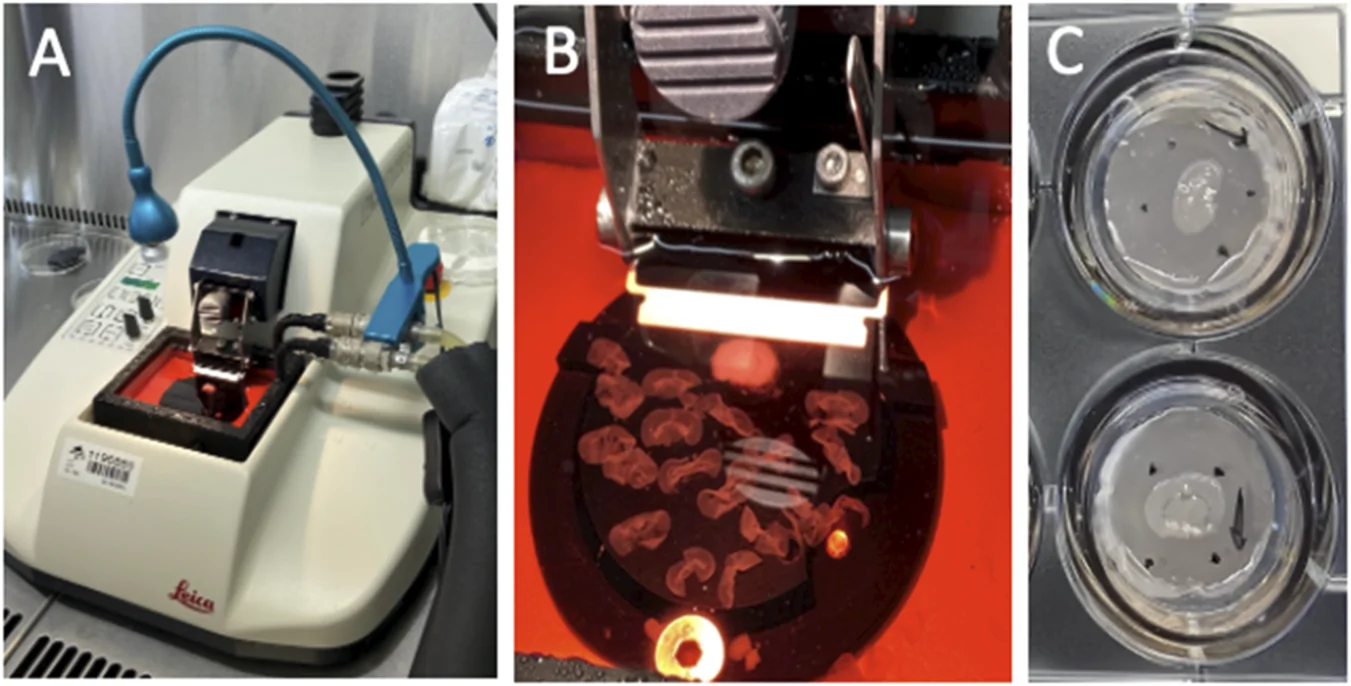
Postnatal (8–10 days) mouse brains were sectioned with a vibratome **(A)** into 150 µm slices. **(B)** The brain slices were cultured on semipermeable membranes in cell culture inserts **(C)**.

Organotypic brain slices were prepared as established in our lab (Weis et al., 2001; Steiner and Humpel, 2021). Briefly, C57BL/6 wildtype mice were decapitated at postnatal days 8–10, and the brains were dissected under sterile conditions. Coronal 150-µm thick brain slices were sectioned at the hippocampal level with a vibratome (Leica Biosystems; Figures 1A,B), and the slices were gently placed on an Isopore™ 0.4-μm pore polycarbonate (PC) membrane (HTTP02500; Merck Millipore, Burlington, Massachusetts, United States; Figure 1C) with or without μCP lanes (see below). These membranes were transferred onto semipermeable 0.4-μm pore cell culture inserts (PICM03050, Merck Millipore), which were placed into 6-well plates (Greiner Bio-One, Kremsmünster, Austria). Each well contained 1.1 ml of sterile-filtered culture medium (50% minimum essential medium/4-(2-hydroxyethyl)-1-piperazineethanesulfonic acid [Gibco, Carlsbad, California, United States], 25% Hanks’ solution [Gibco], 25% heat-inactivated horse serum [Gibco/Lifetech, Austria], 6.5-mg/ml glucose [Merck, Germany], 2-mM NaHCO_3_ [Merck, Austria], and 2-mM glutamine [Merck, Germany] at pH 7.2). The brain slices were cultured at 37 °C with 5% CO_2_ for 2 weeks, and the culture medium was changed once a week. The slices were flattened and became transparent upon attachment to the membranes. After culturing, the slices were fixed for 3 h at 4 °C in 4% paraformaldehyde and then stored at 4 °C in 10 mM of phosphate-buffered saline (PBS) until use. All experiments were conducted in accordance with the Austrian Guidelines for the Ethical Use of Animals and adhered to the 3Rs principle (reduce, refine, and replace), with all efforts made to minimize the number of animals used. All animal experiments were defined as “Organentnahme” according to Austrian laws.

### 2.2 µCP of collagen

µCP was performed as described in our previous studies (Steiner and Humpel, 2021; 2023). The stamps were prepared using a silicon wafer master mold. Briefly, a polydimethylsiloxane (PDMS) prepolymer (SylgardTM 184 Silicone Elastomer Kit, 001004176976; Dow) was gently combined with the elastomer base solution at a ratio of 1:10. The surface relief patterns of the PDMS stamps were produced by casting and curing liquid PDMS onto a micropatterned silicon wafer master mold. This process replicated the raised and recessed features of the silicon wafer onto the stamps, thereby defining the final patterns consisting of 50-µm printing lanes separated by 50-µm spaces or 400-µm large spots. After being left to cure overnight at 60 °C, the solidified PDMS was peeled from the mold, and the stamps were trimmed to size with a scalpel for subsequent use.

For preparation of the collagen load, four-arm star polyethylene glycol succinimidyl succinate (4 S-StarPEG) (JKA7006-1G; Sigma, St. Louis, Missouri, United States) was used as the crosslinker. Sterile bovine collagen solution type I (2 mg/ml, 804,592-20ML; Sigma) was linked to 0.4-mM 4 S-StarPEG in PBS at pH 7.4. As a control, in some experiments red fluorescent Alexa-546 anti-rat antibodies were added at a final concentration of 20 ng/μl in order to check the µCP quality.

For μCP approximately 15 µl of the final collagen solution were applied to each PDMS stamp. To distribute the solution evenly across the stamp surface, a coverslip was gently placed on top, and the stamps were incubated at 37 °C for 15 min. After incubation, the coverslips were removed and gently dragged across the stamp to remove the remaining ink solution and ensure a uniform distribution. Excess solution on the borders of the pattern was removed using filter paper without touching the printing surface. The stamps were left to air dry for a minute. Subsequently, the pre-cut membranes, already placed in wells and coated with 15 µl of polyethylene glycol solution, were stamped with the patterned side facing downwards. To ensure uniform contact, a 4-g weight was applied on top of the stamps, followed by incubation for 1 h at room temperature. The positions of the stamps were marked with four small dots of permanent markers to arrange the slices. The membranes were sterilized under ultraviolet (UV) light for 10 min, equilibrated with the slice medium, and placed on the inserts before arranging the brain slices. For all μCP experiments, we used the Isopore™ 0.4-μm pore PC membrane (HTTP02500; Merck Millipore).

### 2.3 Immunohistochemistry

After fixation, brain slices were stored in PBS containing 0.1% sodium azide at 4 °C. The slices were washed 3x with PBS (1-ml PBS for 3 min). Permeabilization was performed by incubating the slices with 0.1% Triton X-PBS (T-PBS) for 30 min at room temperature on a shaker, followed by three additional PBS washes. Blocking was performed for 30 min at room temperature on a shaker by using 20% horse serum/0.2% bovine serum albumin (BSA)/T-PBS. Following the blocking, the brain slices were incubated in 0.2% BSA/T-PBS with primary antibodies against GFAP (Merck AB5541, 1:2000) or laminin (Sigma L9393; 1:500) for 48 h at 4 °C. After incubation, the brain slices were washed 3x for 3 min each with PBS and incubated with the corresponding green or red fluorescent secondary antibodies (anti-chicken Alexa 488 [Invitrogen A78948.1:400] or anti-chicken Alexa 555 [Invitrogen A78949, 1:300] for GFAP; anti-rabbit fluorescein isothiocyanate [Sigma F9887, 1:200] for laminin) in 0.2% BSA/T-PBS for 1 h at room temperature while shaking. To stain the nuclei, the slices were incubated with 4′,6-diamidino-2-phenylindole (DAPI; 1:10,000 diluted in T-PBS). Finally, the brain slices were washed again with PBS and mounted on glass slides by using Mowiol (Carl Roth, Karlsruhe, Germany). The slides were left to dry and analyzed the following day by using a fluorescence microscope (Olympus BX61, Olympus Corporation, Shinjuku City, Tokyo, Japan) and photographed with a Bresser MicroCam PRO HDMI.

### 2.4 Propidium iodide (PI) staining

To assess cell death, slices were treated with PI. Briefly, fresh living slices were incubated with PI (2 μg/mL) in medium for 1 h at 37 °C. Following incubation, the slices were washed 3x for 3 min each with PBS under gentle shaking and then fixed with paraformaldehyde for 3 h. The fixation step was followed by three washes (3 min each) with PBS, and brain slices were stained using immunohistochemistry.

### 2.5 Live cell imaging of brain slices

Live cell imaging of blood vessels and astrocytes was performed under sterile conditions, as described in detail in our previous work (Humpel, 2025), and we used our novel culture “ring inserts” (Gern et al., 2025) (see Supplementary Figure). Briefly, µCP was used to load collagen lanes onto these “ring inserts” with or without a blue fluorescent (mouse Alexa 350) check antibody, and the inserts were sterilized under UV light and stored at +4 °C until use. On the day of preparation, 150-µm-thick brain slices were sectioned and directly placed on a smaller sterile Isopore™ 0.4-μm pore PC membrane, and µCP was performed on the same day with primary GFAP and laminin antibodies (as described above). Thereafter, a slice (with the membrane) was placed onto a “ring insert” with the µCP side down directly in contact with the collagen lanes (similar to a sandwich) (see Supplementary Figure). These cultures were incubated for 5 days with 1.7-ml medium. The slices were incubated with secondary fluorescent antibodies overnight in medium (chicken red fluorescent for GFAP and rabbit green fluorescent for the vessels [see above]) and then washed in sterile medium. The slices were investigated at days 7, 10, 14, and 17 under an inverted fluorescence microscope with long working distance objectives), with the µCP side facing downward (see Supplementary Figure). The region of interest was selected and photographed, and the slices were immediately transferred to the incubator.

### 2.6 Data analysis and statistics

The optical density (OD) of the cells on each lane was measured. Images of the brain slices were acquired using an Olympus BX61 fluorescence microscope with an objective of ×10, a gain of 45, and an exposure time of 0.09 s. The images were then opened in Adobe Photoshop Elements 2.0 (Adobe, San Jose, California, United States) and converted to grayscale. To ensure comparability across all images, the background intensity of each image was adjusted to 30–35 by regulating the brightness and contrast. Subsequently, a grid was applied to the images to determine the OD on the printed lanes and the spacing between lanes. The OD was determined from a histogram. For each image, six lanes were measured, and the mean values ±standard error of the mean were calculated. For each brain slice, 18 images were captured: 3 from the left hemisphere, and 3 from the right hemisphere. For the linear size measurements, a picture from the slice was made at a ×20 magnification together with a calibrated scale marker, and the size of the vessels or GFAP lanes or dots was analyzed using ImageJ. For each analysis at least 6–10 areas were measured and averaged. Statistical analysis was performed using one-way analysis of variance with a subsequent Fisher’s least significant difference *post hoc* test, where p < 0.05 represents statistical significance. In all data sets the value n represents the number of independent mice. In all cases n = 1 represents the average of at least 6–10 independent measurements in 1 slice.

## 3 Results

### 3.1 Laminin restructuring

**FIGURE 2:**
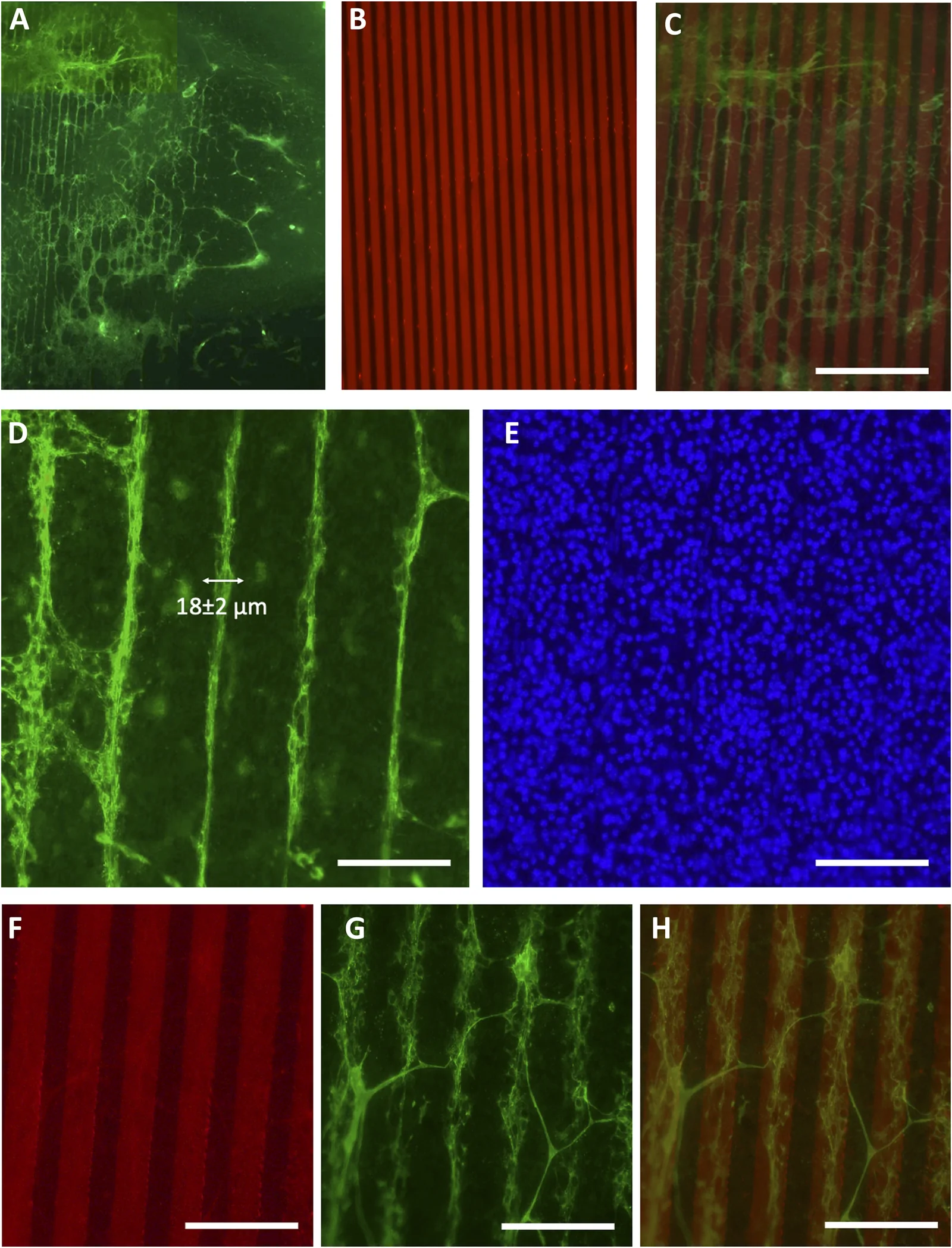
Organotypic brain slices **(A)** were cultured on collagen-loaded lanes **(B)** as shown in a merged picture **(C)** for 2 weeks. Laminin^+^ vessels (green fluorescent with Alexa-488) re-organized with a thickness of 18 ± 2 µm on the lanes **(D)** which does not alter the blue fluorescent nuclear DAPI layer **(E)**. A co-localization **(H)** shows that the red fluorescent collagen lanes **(F)** are closely associated with the laminin^+^ vessels **(G)** Scale bar = 1,150 µm **(A)** 400 µm **(B)** 290 µm **(C)** 130 µm **(D,E)** 230 µm **(F–H).**

Laminin^+^ vessels underwent marked restructuring along 50-µm-wide collagen microcontact prints and formed long, straight vascular structures that elongated throughout the brain slice. The laminin^+^ vessels reached a length of 2062 ± 194 µm (n = 4 mice) (Figures 2A–C). The reorganized vessels aligned with the collagen lanes (Figures 2F–H) and exhibited a thickness of 18 ± 2 µm (n = 4 mice) (Figure 2D) and did not alter the DAPI layer (Figure 2E).

### 3.2 GFAP restructuring

**FIGURE 3:**
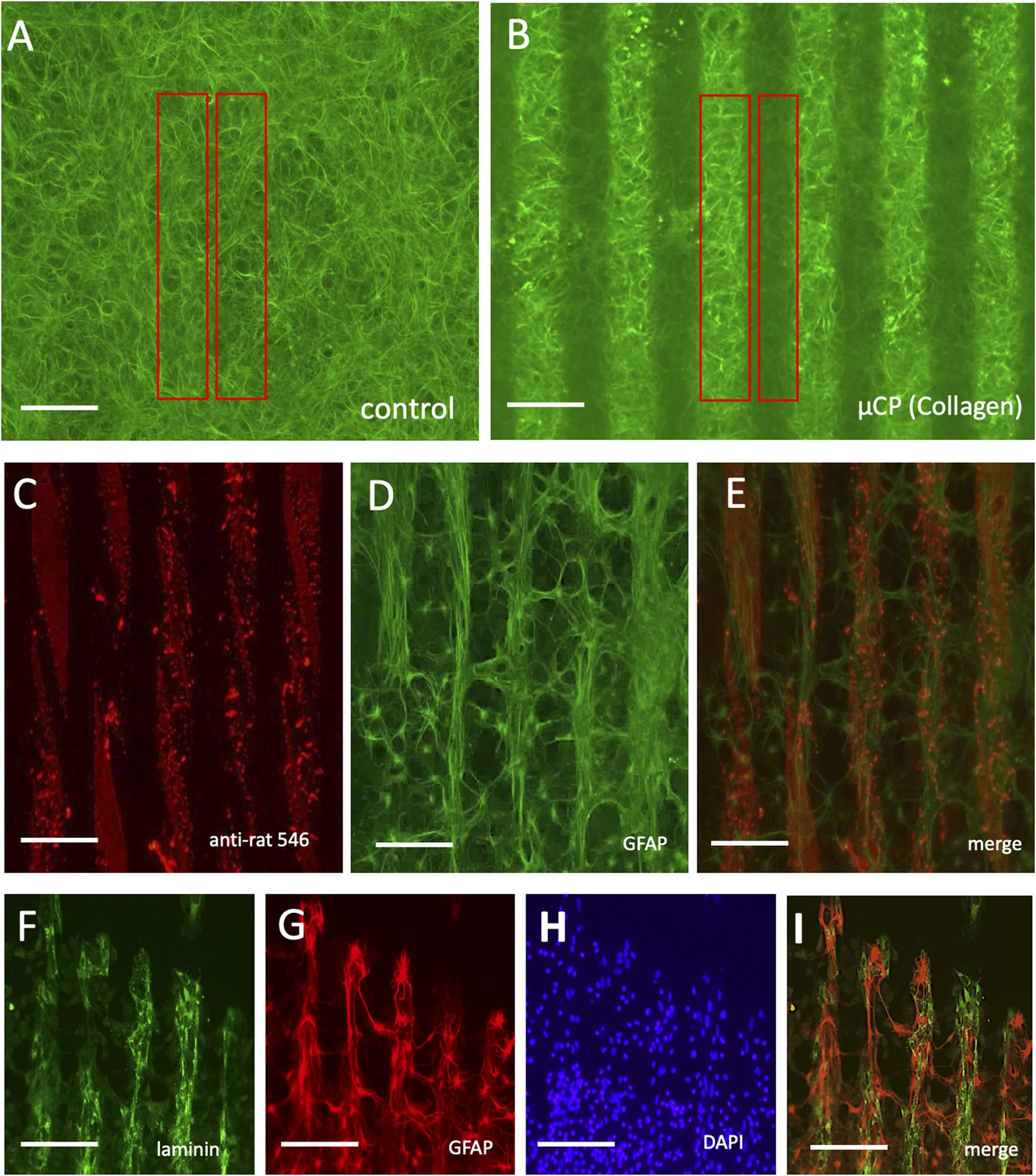
Reorganization of glial fibrillary acidic protein (GFAP) along the microcontact printed (µCP) collagen printed lanes and interaction with laminin after 2 weeks in culture. Compared to a control without collagen lanes **(A)** green fluorescent Alexa-488 GFAP^+^ astrocytes restructured on collagen-coated lanes **(B)** Co-localization **(E)** shows that the green fluorescent GFAP^+^ astrocytes reorganized **(D)** directly on the red labelled collagen lanes **(C)** Co-localization **(I)** shows that the green fluorescent (Alexa-488) laminin lanes **(F)** are closely associated with the red fluorescent (Alexa-546) astroglia lanes **(G) (H)** shows the blue fluorescent nuclear DAPI layer in the same area. Scale bar = 80 µm **(A,B)** 115 µm **(C–E)** 175 µm **(F–I).**

Similar to laminin, GFAP^+^ astroglia also restructured on the microcontact-printed collagen lanes (Figure 3). Compared with the controls (Figure 3A), dense lanes of GFAP^+^ (58.2 ± 0.6 OD, n = 9 mice) and GFAP− (36.2 ± 0.9 OD, n = 9 mice, ***) lanes were observed (Figure 3B). The size of the GFAP lanes was 48.5 ± 0.6 µm (n = 6 mice).

### 3.3 Restructuring on large 400-µm collagen spots

**FIGURE 4:**
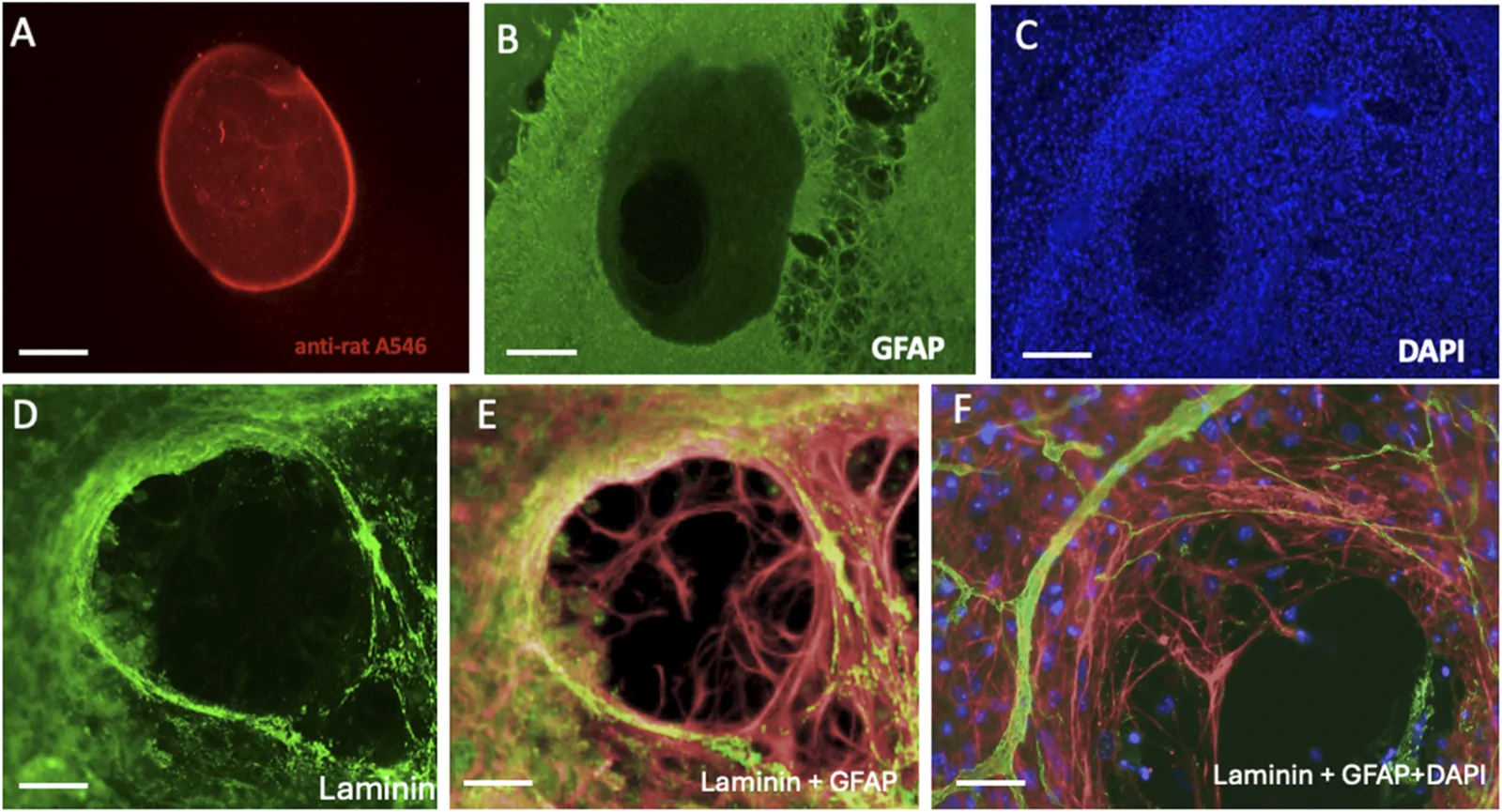
Effects of large 400 µm collagen spots after culture for 2 weeks. Round collagen spots are visualized with a red fluorescent anti-rat antibody. **(A)** After culture a large weak area of green-fluorescent (Alexa-488) glial fibrillary acidic protein (GFAP) staining is seen around the collagen spots **(B)** with some DAPI negative areas. **(C)** These DAPI negative areas are surrounded by green fluorescent (Alexa-488) laminin + vessels **(D)** and red fluorescent (Alexa-546) GFAP^+^ processes **(E)** growing into the DAPI negative **(F)** area. Scale bar = 260 μm **(A)** 260 µm **(B,C)** 100 µm **(D–F).**

We expected the same effects on large, round 400-µm collagen spots as those on 50-µm-wide lanes. The spots were visualized using an anti-red fluorescent Alexa-546 check antibody with a size of 868±19 μm (n = 6 mice) (Figure 4A). Collagen µCP caused a significantly decreased GFAP staining with the same size (n = 6 mice) (Figure 4B). A closer inspection revealed some DAPI negative areas (Figure 4C) within these spots with a size of 99±31 μm. Among the 60 collagen spots analyzed, only 28 (47%) showed a clear change in GFAP staining, and only 13 showed DAPI-negative staining. Almost no vessels were observed in these DAPI-negative holes, but there were some astroglial processes and a stronger vessel density around the holes (Figures 4D–F).

### 3.4 PI^+^ cell death

**FIGURE 5:**
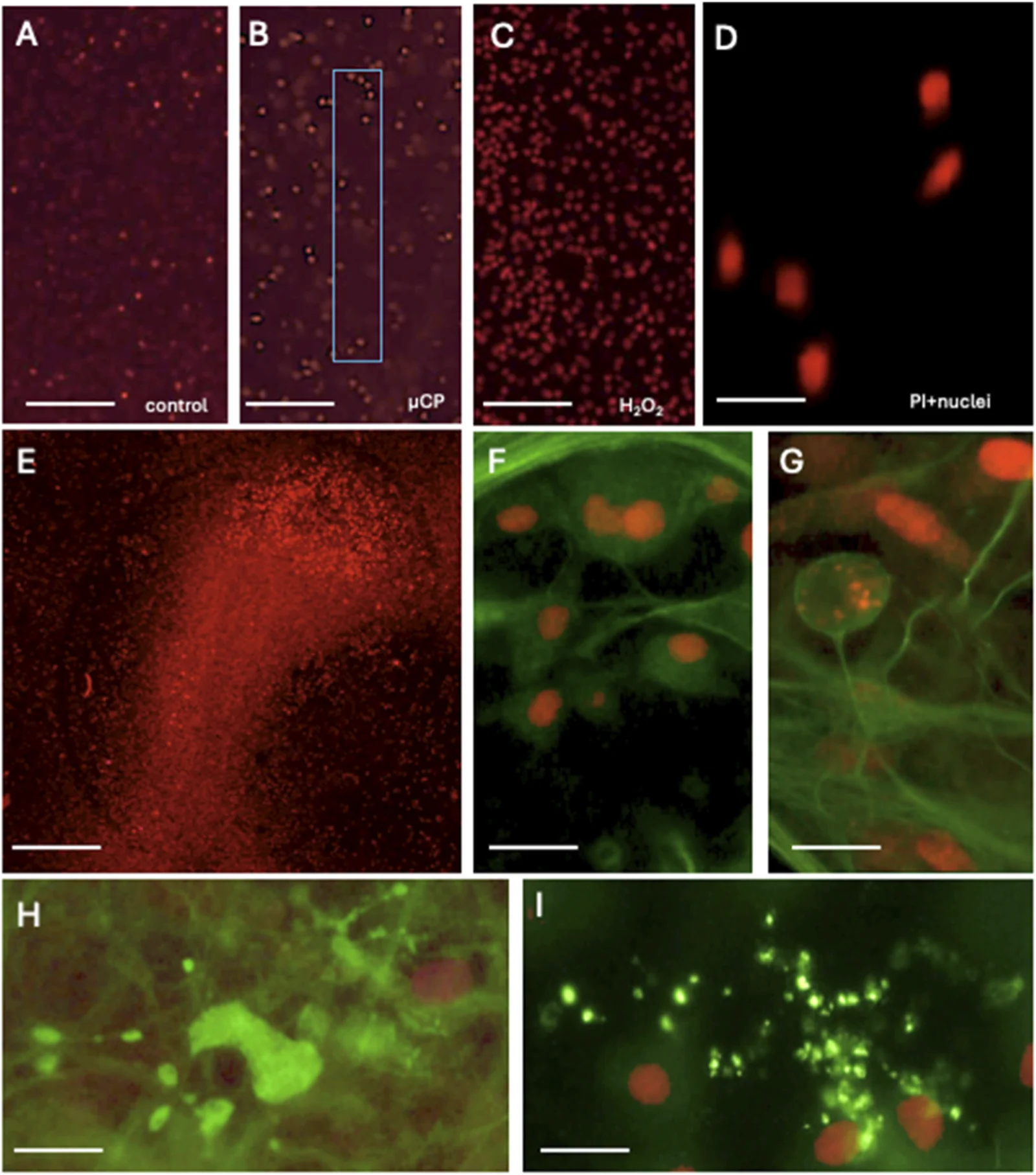
Cell death of astrocytes in the microcontact printed (µCP) collagen spots. Brain slices were cultured on collagen spots for 2 weeks and then stained for propidium iodide (PI). **(A)** shows a control without any collagen. **(B)** shows a slice cultured on lanes (a lane is indicated by a blue box) and **(E)** shows slices cultured on spots. As a control, slices were treated with peroxide (H_2_O_2_). **(C)** A higher magnification shows red fluorescent PI^+^ nuclei. **(D)** Co-localization of red fluorescent PI^+^ nuclei (>1,500 per mm^2^) with green fluorescent (Alexa-488) glial fibrillary acidic protein (GFAP)+ astroglia shows several types of astroglial cell death in the collagen holes **(F–I).** Scale bar = 80 µm **(A–C)** 10 µm **(D)** 150 µm **(E)** 30 µm **(F–I).**

PI staining was performed to observe cell death as the red fluorescent PI dye easily penetrates dying cells and stains nuclei (Figure 5D). In control slices without collagen, very few PI^+^ nuclei were observed (396 ± 35 per mm^2^, n = 7 mice) (Figure 5A). As a control hydrogen peroxide significantly enhanced the number of PI^+^ nuclei (2,156 ± 207 per mm^2^, n = 7 mice; p < 0.001). (Figure 5C). Slightly (but not significantly) enhanced PI^+^ nuclei were observed in the slices cultured on collagen lanes (766 ± 134 per mm^2^, n = 9 mice). (Figure 5B). In slices with 400-µm printed collagen spots, we found areas with low but also with very high (>1,500 per mm^2^) PI^+^ nuclei (Figure 5E), but they were not quantified.

To further characterize the GFAP^+^ astrocytes in these very high (>1,500 per mm^2^) PI^+^ areas, a costaining with GFAP was performed. Immunostaining revealed that many GFAP^+^ astrocytes showed signs of cell death either with weaker GFAP staining and reduced processes (Figure 5F), a round shape with fragmented PI^+^ nuclei (Figure 5G), very strong GFAP cytoplasm and degenerated shape (Figure 5H), or extremely fragmented astrocytes with fragmented processes (Figure 5I).

### 3.5 Laminin^+^ vessels and GFAP^+^ astroglia in 6-week-old cultures

**FIGURE 6:**
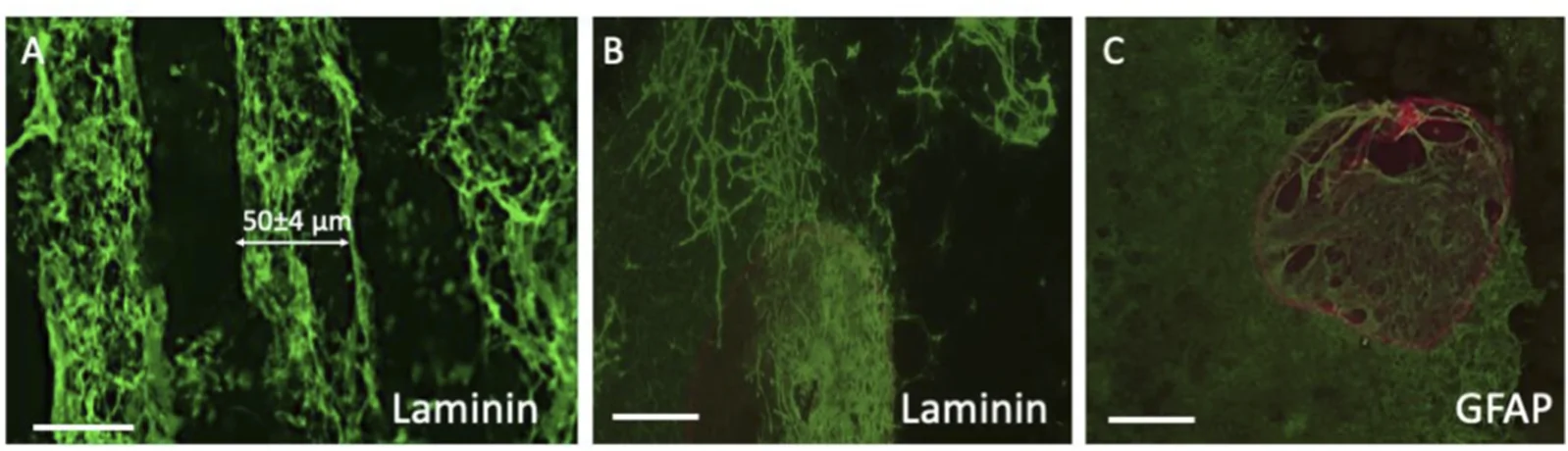
Reorganization after 6 weeks in culture. laminin^+^ vessels **(A,B)** and glial fibrillary acidic protein (GFAP)+ astroglia **(C)** were cultured on collagen lanes **(A)** or red labelled collagen spots **(B,C)** for 6 weeks. Note that laminin + vessels were larger on collagen lanes after 6 weeks, while vessels **(B)** as well as glial processes **(C)** grow into the collagen spots after 6 weeks. Scale bar = 40 µm **(A)** 160 µm **(B,C).**

When slices were cultured for 6-week on collagen lanes, very dense laminin^+^ networks were formed (Figure 6A) with a width of 50 ± 4 µm (n = 3 mice), which were significantly (p < 0.001) thicker than 2-week-old cultured slices (Figure 2D) with a thickness of 18 ± 2 µm (n = 4 mice) (***). After 6 weeks of culturing on collagen spots, the slices exhibited clear signs of vasculogenesis, which is characterized by the prominent ingrowth of a thin, cross-linked vascular network extending beyond the edge of the spot (Figure 6B). When slices were cultured for 6-week on collagen spots, the DAPI-negative areas within those slices were slightly, but not significantly, higher (18 ± 5%, n = 4 mice) than the 2-week-old slices (11 ± 4%, n = 9 mice). Interestingly, although the number of cell bodies within the spots was reduced, several GFAP^+^ cell processes and a thick GFAP^+^ fiber network were visible even in DAPI-negative areas (Figure 6C).

### 3.6 Time-dependent restructuring of laminin–GFAP on lanes and spots

**FIGURE 7:**
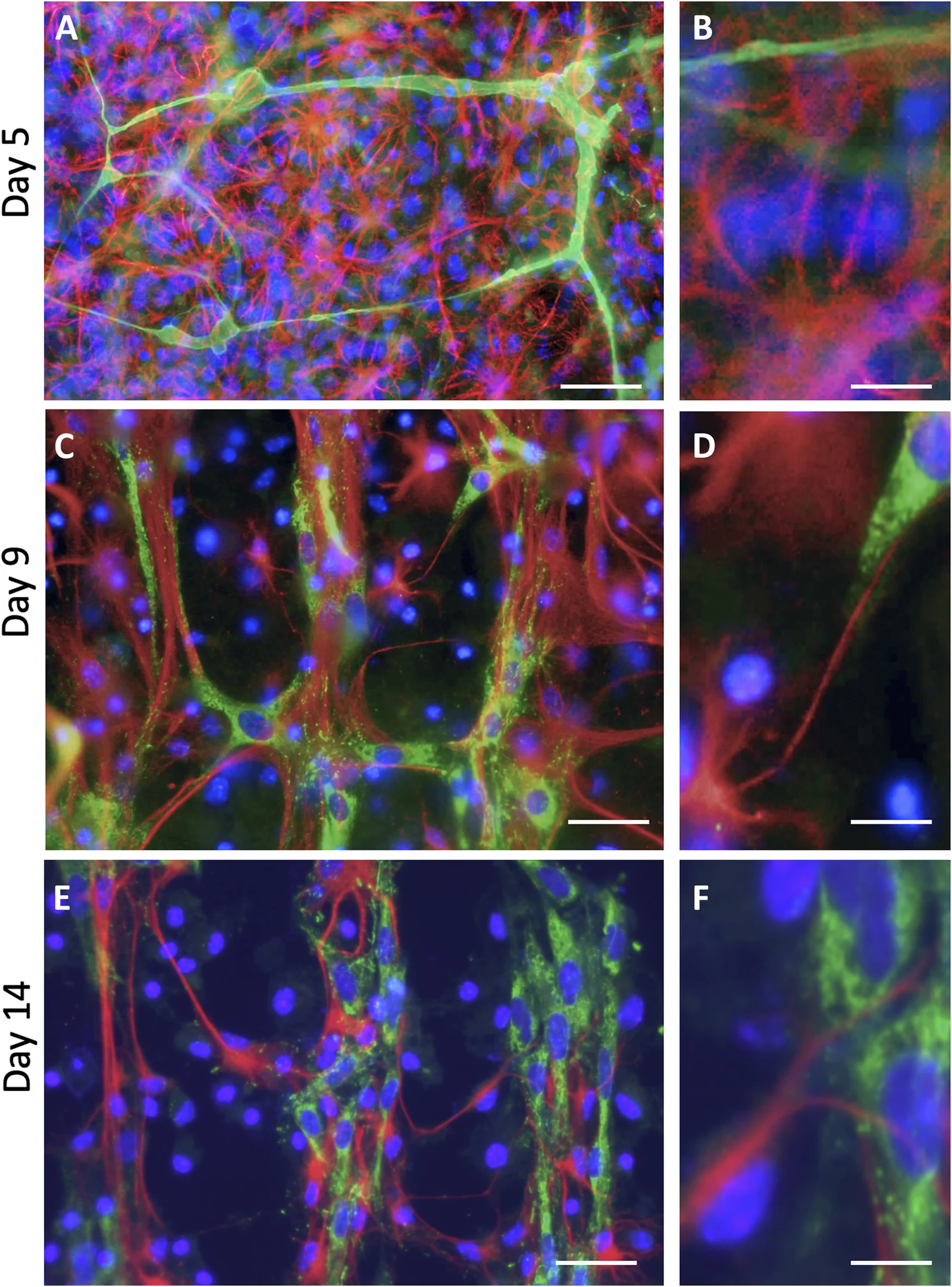
Time dependent restructuring after 5 **(A,B)**, 9 **(C,D)** and 14 **(E,F)** days cultured on collagen lanes. The pictures show co-localization of green fluorescent (Alexa-488) laminin + vessels and red fluorescent (Alexa-546) glial fibrillary acidic protein (GFAP)+ astrocytes counterstained with blue-fluorescent DAPI. Note that the restructuring starts after 6 days of culture and reaches full restructuring after 14 days with new astroglial processes on vessels. Scale bar = 35 µm **(A,C,E)** and 10 µm **(B,D,F)**.

To observe the time-dependent restructuring of laminin and GFAP on the collagen lanes, a high-power image was taken after 5, 9, and 14 days. After 5 days, there was little or no restructuring along the lanes (Figures 7A,B). The restructuring was in full progress on day 9 (Figures 7C,D) and was complete after 14 days (Figures 7E,F). Interestingly, new astroglial contacts formed on the laminin^+^ vessels on day 14 (Figure 7F).

**FIGURE 8:**
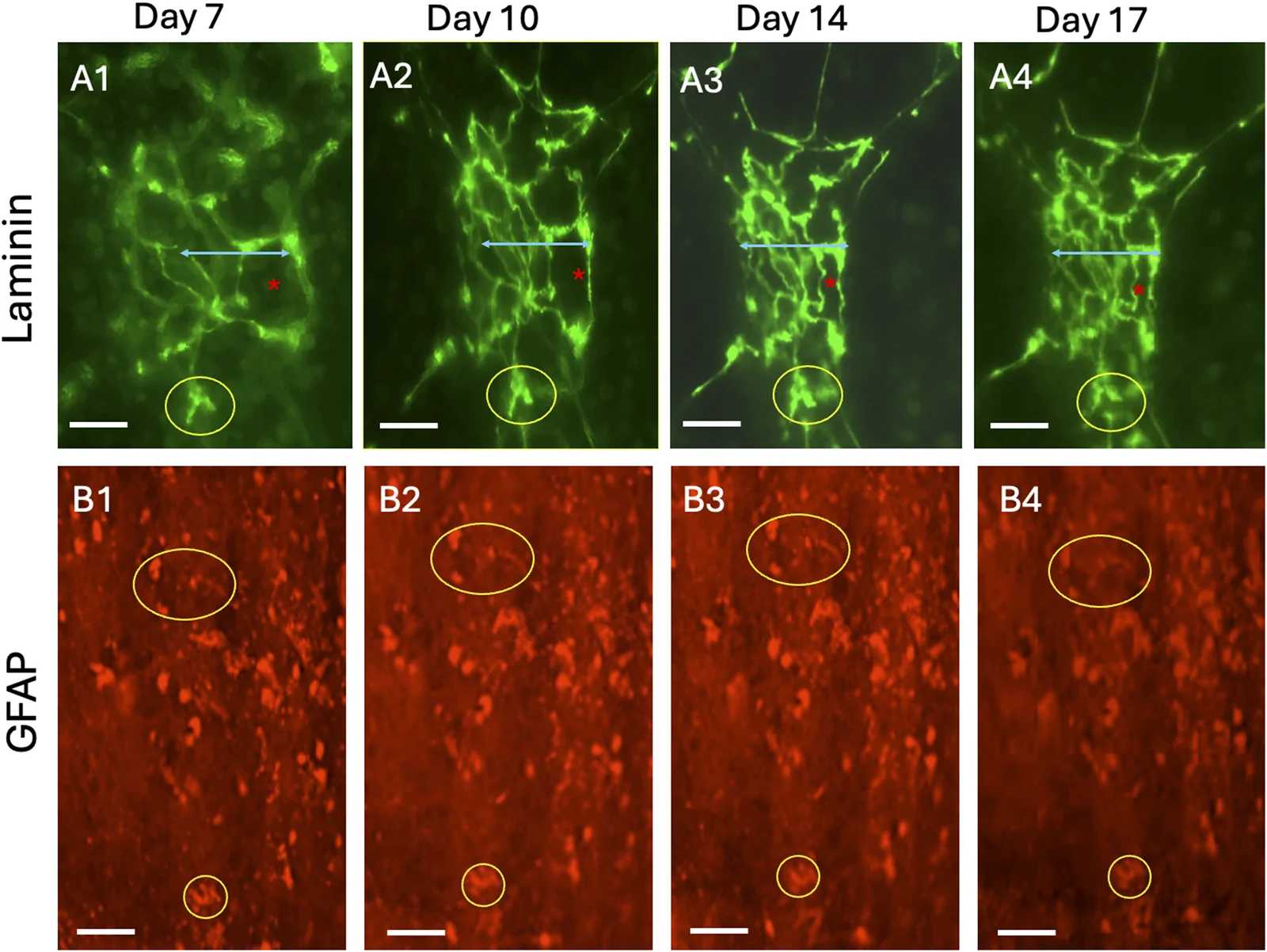
Live cell imaging was performed on our novel “ring-inserts” (see Supplementary Figure and Gern et al., 2025 for details). Our preliminary/exploratory data show that laminin^+^ vessels **(A)** as well as glial fibrillary acidic protein (GFAP)+ astrocytes **(B)** can be followed up on day 7 (A1, B1), day 10 (A2, B2), day 14 (A3, B3) and day 17 (A4, B4). Some laminin^+^ vessels became thinner after day 10, while GFAP + astrocytes seemed to re-structured before day 7. The yellow circles in **(A)** (vessel) and **(B)** (astrocytes) point to the same areas, the red star labels the same vessel structure and the length of the blue arrow-line is identical in all pictures **(A)** (vessel). Scale bar = 60 µm **(A,B)**.

To follow up the time-dependent effects, live cell imaging was used on slices cultured on our new modified “ring inserts” (for details see the Supplementary Figure). Slices cultured on the ring inserts were healthy and vessels and astrocytes reorganized on the blue fluorescent collagen lanes (Supplementary Figure). Live cell imaging was very difficult and only partially successful and reproducible because of the complexity of the setup. Our preliminary/exploratory data showed that some laminin^+^ vessel networks became thinner after 10–14 days of culture (Figures 8A1–A4). Our preliminary/exploratory data also showed that GFAP^+^ astrocytes reorganized before the vessels on day 7 and did not change for up to 17 days (Figures 8B1–B4).

## 4 Discussion

In this study, we showed that collagen can induce the restructuring of astrocytes and vessels in organotypic brain slices when grown on 50-µm-thin collagen lanes. This time-dependent process began on day 5 and ended on day 14. By contrast, when slices were exposed to a larger area of 400 µm collagen spots, GFAP was reduced accompanied with cell death of astrocytes.

### 4.1 Organotypic brain slices and viability

We established organotypic brain slices for research purposes 25 years ago and have since used them in many experiments (Humpel, 2025). The brain slices survived well, remained healthy under defined conditions, and could be cultured for several weeks. First, a slice thickness of 150 µm proved to be optimal for survival and later visualization. Second, the age of the donor animal is a critical factor that influences the quality of organotypic brain slices. In our laboratory, we used postnatal day 8–10 mice because slices obtained at this developmental stage exhibit superior morphology and viability compared with slices from older donors. The quality of the slices can be validated by their visual appearance, given that viable slices fully attach to the membrane, flatten, and become more transparent. Viability can also be verified at the end of the culture by using histological staining with DAPI (Tarnowski et al., 1991), which mainly shows a clear homogeneous cell layer over the brain slice. Additionally, cell death assays with PI are performed on living tissue slices, which do not result in many red fluorescent nuclei in the brain tissue. PI selectively stains dead cells because it cannot penetrate an intact plasma membrane (Crowley et al., 2016). More PI-positive nuclei were observed only at the edges of the slices, indicating scar formation at the edges. Finally, immunohistochemical staining shows the ultimate viability of brain cells. Considering that we incubated free-floating slices, the antibody could penetrate from both sides. Our model also allows for the costaining of different structures with several fluorescent dyes (blue–green–red fluorescence) to provide insights into 3D tissue organization.

### 4.2 Collagen and astrocytes in the brain

In this study, we showed that collagen markedly reorganizes astrocytes when grown on collagen lanes. We reproduced and extended our previous study (Steiner and Humpel, 2021) and showed the rapid reorganization of astrocytes in organotypic brain slices between days 5 and 14. Although our study is the first to show such a strong reorganization of astrocytes on collagen lanes, our result is in agreement with other studies showing that collagen can bind to astroglial integrins and activate the integrin–N-cadherin signaling pathway (Hara et al., 2017). The activation of this pathway promotes the transformation of reactive astrocytes into scar-forming astrocytes, thus restricting axonal regeneration and functional recovery (Hara et al., 2017). Moreover, the activation of the integrin signaling cascade enhances astrocytic cell–cell adhesion by upregulating N-cadherin expression (Camand et al., 2012). In the current study, we did not elucidate the exact cellular mechanism of collagen; however, it is possible that the patterned collagen interacts with astrocytic integrins and activates the integrin–N-cadherin signaling pathway. In future studies, we plan to block the integrin pathway and expect to inhibit this reorganization and reduce scar formation, as suggested previously (Hara et al., 2017).

### 4.3 Collagen and brain vessels

We showed that collagen not only restructures astrocytes but also induces the reorganization of laminin^+^ vessels along collagen lanes. Collagen contributes to BBB stability and plays an important role in cellular organization and morphogenesis (Singh et al., 2012). In addition to its structural function, collagen is involved in maintaining the balance between neuronal, glial, and vascular components under conditions of cellular stress. Chronic or excessive cellular stress can cause increased collagen deposition and enhanced crosslinking within the ECM, thus resulting in matrix stiffening (Wareham et al., 2024). By contrast, inflammatory processes cause collagen degradation, which also contributes to vascular damage (Schwarz et al., 2022) and the increased sensitivity of endothelial cells to interleukin-1 (Weber et al., 2010). The activation of interleukin-1 in damaged vessels potentiates inflammatory signaling and promotes vascular dysfunction. Moreover, after inflammation or injury, the ECM facilitates the adhesion of endothelial cells to collagen and contributes to vascular remodeling (Summers et al., 2013). We did not show the exact mechanism of vascular reorganization but favor the idea that the inflammatory processes caused by glial activation may contribute to this collagen-mediated effect.

### 4.4 Timeframe of astroglial and vessel reformation

Astrocytes and blood vessels interact closely in the brain, with astrocytes extending processes toward vessels to regulate vascular function (Hösli et al., 2022; Koehler et al., 2006). Thus, we aimed to follow up these effects by using our novel “ring inserts” (Humpel, 2025; Gern et al., 2025; see also Supplementary Figure), where we could clearly show the collagen-mediated restructuring. For live cell imaging, living astrocytes and vessels in the slices were stained with primary antibodies, cultured for 5 days, and labeled with fluorescent antibodies (green fluorescence for vessels and red fluorescence for astrocytes). By using this method, we were able to follow cells in living brain slices for several weeks. This technically demanding procedure involves transferring the slices to a microscope under sterile conditions, identifying and imaging the area of interest, and immediately returning the slices to the incubator. On the basis of our data, we aimed to photograph the labeled cells on days 7–14 and 17 by using a microscope. A key challenge in this regard is the coupling of labeled brain slices to collagen lanes. To address this, we used µCP with freshly dissected slices, and coupled them directly to collagen lanes on the “ring inserts.” (see Supplementary Figure). However, the live cell imaging of µCP-labeled astrocytes and vessels is complex and inconsistent. Nevertheless, our preliminary/exploratory data show that astrocytes may have reorganized before the vessels on day 7, and vessels more likely showed a tendency to shrink together and become more closely connected. The causes for low reproducibility remain unclear, but we propose several possibilities: (1) µCP may induce cellular stress, thus leading to reduced functional activity, possibly involving calcium dysregulation. (2) Labeling with large primary and fluorescent antibodies may impair function, for example, by interfering with collagen receptor signaling. (3) Slice thickness and/or µCP may limit the direct contact between labeled cells and collagen lanes and/or may delay slice flattening. We are currently optimizing this live cell imaging approach to achieve better cellular resolution and reproducibility. But again, we clearly state that these are preliminary/exploratory data.

### 4.5 Collagen and cell death

Collagen has positive effects on the restructuring of brain cells. However, we also show that collagen has negative cell death–promoting effects under certain conditions. It is assumed that an appropriate interaction between the cell membrane and ECM contributes to cellular stabilization and differentiation. By contrast, impaired cell–matrix interactions may, under certain conditions, trigger apoptotic pathways, which is referred as “disoriented cell death.” Studies have shown that cells undergoing structural reorganization tend to die at later stages. This may indicate that the remodeling process compromises the ability of the cell to maintain or re-establish optimal interactions with the ECM, thus ultimately leading to cell death (Tang et al., 1998). However, the exact mechanism underlying collagen-induced apoptosis has not yet been fully elucidated (Tang et al., 1998; Nho and Hergert, 2014). Although it can occur in chondrocytes (Cao et al., 1999) or neural stem cells (O’Connor et al., 2001; Nakaji-Hirabayashi et al., 2013), it has been demonstrated that collagen-induced cell death only occurs in polarized cells. Furthermore, signaling via α2-integrin localized at the apical membrane was shown to be required for the induction of apoptosis (Tang et al., 1998). Additionally, collagen undergoes structural alterations during natural aging (Wilson et al., 2012). These age-related modifications may impair cell–matrix interactions, thus potentially disrupting essential adhesion-mediated signaling pathways and contributing to the induction of apoptosis. Moreover, collagen production declines with age, which may further compromise cell–matrix interactions. Reduced collagen availability can impair structural support and adhesion-mediated signaling, thereby increasing cellular susceptibility to apoptosis (Wareham et al., 2024). In the present study we show that a larger area of collagen (400 µm) can cause cell death while on smaller areas (50 µm) reformation occurs. This is not caused by a higher dose of collagen, as the method is identical to apply the collagen, but rather to the larger area, which may cause a signalling in the brain simulating a large brain insult. However, the exact mechanism we could not explore so far.

### 4.6 Collagen-induced restructuring in aging and AD

AD is a neurodegenerative disorder and is primarily driven by the abnormal accumulation of misfolded beta-amyloid (Flier et al., 1991; Walker, 2020) and tau proteins (Kolarova et al., 2012). In addition to these depositions in the brain, cholinergic neuronal degeneration and neuroinflammation are initiated, accompanied by the activation of microglia and astrocytes (Reale and Costantini, 2021). Elevated concentrations of collagen have been observed in AD, and this upregulation is associated with increased collagen deposition in the brain, possibly contributing to vascular dysfunction (Anwar et al., 2022). In addition, collagen interacts with beta-amyloid and facilitates clearance and neuroprotective effects (Ma et al., 2020). By contrast, collagen dysfunction impairs brain function, ultimately inhibiting neuronal repair and regeneration (Nimni, 2018; Wareham et al., 2024). This dysfunction could be caused by matrix metalloproteinases, which are expressed and released from reactive astrocytes and may promote the degradation of collagen (Rabkin, 2023). This clearly supports our findings that collagen has protective and regenerative activities in aging and degenerating brains, but can also cause cell death when becoming dysfunctional.

### 4.7 Limitations

This study has a few limitations that should be considered when interpreting the results. (1) Organotypic brain slices represent valuable *in vitro* models that preserve much of the complex 3D architecture of the brain. However, they cannot fully replicate the exact anatomical and physiological conditions of the intact brain *in vivo* because the preparation of slices inevitably disrupts blood circulation, thereby eliminating the physiological vascular supply. Consequently, the translation of *in vitro* findings to *in vivo* conditions remains limited. (2) Another limitation is the age of the donor tissue. Given that AD is an age-related neurodegenerative disease, postnatal brain slices do not optimally reflect the pathological environment of the aged brain. Although adult mouse brain slices would be more appropriate in this regard, they are technically challenging to culture because of their reduced cell viability and limited ability to flatten and stabilize compared with postnatal slices (Humpel, 2015; Croft et al., 2019). (3) It is not possible to adequately model all AD pathologies in organotypic brain slices (Humpel, 2025), thus limiting the interpretation of the disease. Moreover, AD develops over several years, whereas organotypic brain slices can only be maintained in culture for a few weeks. This relatively short cultivation period may not have been sufficient to fully observe long-term astrocytic and vascular alterations. (4) The applicability of these results to the human brain may be limited because the experiments were conducted using murine brain slices. However, mice and humans share a high degree of genetic and physiological homology, thus suggesting that these findings are likely relevant despite potential species-specific differences. (5) There are also technical limitations associated with the preparation and maintenance of organotypic brain slices. The establishment and cultivation of viable and reproducible slice cultures requires substantial technical expertise, and minor variations in handling may affect experimental outcomes. Despite these challenges, all procedures were performed carefully to ensure consistency and reliability. (6) In addition, live cell imaging is very complex and challenging because the daily transfer of slices and their visualization under a microscope for 20–30 min are very sensitive procedures. In this context, we could not wash the slices extensively, leading to a very high background under the fluorescence microscope. Furthermore, the thickness of the slices did not allow for the creation of perfectly sharp images.

### 4.8 Future implications

Future studies should (based on our preliminary/exploratory data) investigate the long-term interactions between astrocytes and blood vessels. By placing the membrane insert in a controlled incubation chamber mounted directly on the microscope, the repeated transfer of slices would no longer be necessary. This setup would allow for the continuous observation of the same brain region, thus enabling the generation of video recordings and reducing the variability caused by repositioning.

In addition, it is important to establish a method that allows for the examination of both postnatal and adult mouse brain slices. The inclusion of adult tissues would reflect age-related processes more closely and could provide more precise and reliable insights into the simulation of the aging human brain. Another interesting direction is to examine the effects of collagen inhibition on astrocytic and vascular functions. Collagen expression can be reduced using antisense oligonucleotides that specifically target collagen messenger RNA, thereby suppressing translation (Jorfi et al., 2018). Alternatively, a transgenic mouse model could be employed in which collagen-related pathways are genetically modified. Furthermore, the use of transgenic mice expressing human Aβ or tau protein would allow the investigation of AD-related mechanisms in combination with altered ECM composition. If these approaches could be successfully established, they could substantially improve the translational relevance of AD research because experimental findings would better reflect the pathological processes occurring in the human brain. In this context, it would be interesting to perform similar experiments by using *postmortem* human brain slices derived from patients with AD. However, further methodological developments are required because it is not fully understood how human brain slices can be reliably cultured and maintained *in vitro* over extended periods of time (Jorfi et al., 2018).

## 5 Conclusion

These findings highlight the dual role of collagen in regulating cell survival and providing a neuroregenerative function. The conditions under which collagen triggers cell death remain unclear.

## Data Availability

The raw data supporting the conclusions of this article will be made available by the authors, without undue reservation.

## Supplementary material

SUPPLEMENTARY FIGURE Live cell imaging was performed on our novel modified ring inserts (see Gern et al., 2025). Rings with a diameter of 20 mm were prepared **(A)**. Slices were prepared and placed on the rings (red dotted line), then microcontact printed and labelled with fluorescence antibody and cultured in 1.7 mL medium for 5 days **(B)**. Next an extra membrane (green dotted line) with the printed collagen lanes (blue boxes) was placed directly onto the slice (like a sandwich) **(B)** and cultured **(C)**. For live cell imaging the ring with the slice was taken, turned and investigated inverse in the fluorescence microscope **(D)**. Laminin^+^ vessels **(E)** as well as glial fibrillary acidid protein + astroglia **(F)** reorganize on the blue fluorescent (Alexa 350) collagen loaded rings **(G)**. Scale bar = 100 µm **(E–G)**.

## References

[B1] Anwar M. M. Özkan E. Gürsoy‐Özdemir Y. (2022). The role of extracellular matrix alterations in mediating astrocyte damage and pericyte dysfunction in Alzheimer’s disease: a comprehensive review. Eur. J. Neurosci. 56, 5453–5475. 10.1111/ejn.15372 34182602

[B2] Brenner M. Messing A. (2021). Regulation of GFAP expression. ASN Neuro 13, 1759091420981206. 10.1177/1759091420981206 33601918 PMC7897836

[B3] Brightman M. (1991). Implication of astroglia in the blood‐brain barrier. Ann. N. Y. Acad. Sci. 633, 343–347. 10.1111/j.1749-6632.1991.tb15625.x 1789557

[B4] Camand E. Peglion F. Osmani N. Sanson M. Etienne-Manneville S. (2012). N-cadherin expression level modulates integrin-mediated polarity and strongly impacts on the speed and directionality of glial cell migration. J. Cell Sci. 125, 844–857. 10.1242/jcs.087668 22275437

[B5] Cao L. Lee V. Adams M. E. Kiani C. Zhang Y. Hu W. (1999). β1-Integrin–collagen interaction reduces chondrocyte apoptosis. Matrix Biol. 18, 343–355. 10.1016/S0945-053X(99)00027-X 10517181

[B6] Cohen‐Salmon M. Slaoui L. Mazaré N. Gilbert A. Oudart M. Alvear‐Perez R. (2021). Astrocytes in the regulation of cerebrovascular functions. Glia 69, 817–841. 10.1002/glia.23924 33058289

[B7] Croft C. L. Futch H. S. Moore B. D. Golde T. E. (2019). Organotypic brain slice cultures to model neurodegenerative proteinopathies. Mol. Neurodegener. 14, 45. 10.1186/s13024-019-0346-0 31791377 PMC6889333

[B8] Crowley L. C. Scott A. P. Marfell B. J. Boughaba J. A. Chojnowski G. Waterhouse N. J. (2016). Measuring cell death by propidium iodide uptake and flow cytometry. Cold Spring Harb. Protoc. 2016, prot087163. 10.1101/pdb.prot087163 27371595

[B9] Flier J. S. Underhill L. H. Yankner B. A. Mesulam M.-M. (1991). β-Amyloid and the pathogenesis of alzheimer’s disease. N. Engl. J. Med. 325, 1849–1857. 10.1056/NEJM199112263252605 1961223

[B10] Gern A. Lehmann P. Schäfer J. Humpel C. (2025). Organotypic mouse brain slices: low-cost “ring-inserts” to study cholinergic and dopaminergic neurons with live cell imaging with an emphasis on calcium imaging. Biofunctional Mater. 3 (2). 10.55092/bm20250011

[B11] Halder S. K. Sapkota A. Milner R. (2023). The importance of laminin at the blood-brain barrier. Neural Regen. Res. 18, 2557–2563. 10.4103/1673-5374.373677 37449589 PMC10358660

[B12] Hara M. Kobayakawa K. Ohkawa Y. Kumamaru H. Yokota K. Saito T. (2017). Interaction of reactive astrocytes with type I collagen induces astrocytic scar formation through the integrin–N-cadherin pathway after spinal cord injury. Nat. Med. 23, 818–828. 10.1038/nm.4354 28628111

[B13] Hatten M. E. Liem R. K. H. Shelanski M. L. Mason C. A. (1991). Astroglia in CNS injury. Glia 4, 233–243. 10.1002/glia.440040215 1827781

[B14] Hol E. M. Pekny M. (2015). Glial fibrillary acidic protein (GFAP) and the astrocyte intermediate filament system in diseases of the central nervous system. Curr. Opin. Cell Biol. 32, 121–130. 10.1016/j.ceb.2015.02.004 25726916

[B15] Hösli L. Zuend M. Bredell G. Zanker H. S. Porto De Oliveira C. E. Saab A. S. (2022). Direct vascular contact is a hallmark of cerebral astrocytes. Cell Rep. 39, 110599. 10.1016/j.celrep.2022.110599 35385728

[B16] Humpel C. (2015). Organotypic brain slice cultures: a review. Neuroscience 305, 86–98. 10.1016/j.neuroscience.2015.07.086 26254240 PMC4699268

[B17] Humpel C. (2018). Organotypic brain slice cultures. Curr. Protoc. Immunol. 123, e59. 10.1002/cpim.59 30311739

[B18] Humpel C. (2025). Long-term live-cell imaging of GFAP+ astroglia and laminin+ vessels in organotypic mouse brain slices using microcontact printing. Front. Cell. Neurosci. 19, 1540150. 10.3389/fncel.2025.1540150 39935610 PMC11808140

[B19] Jorfi M. D’Avanzo C. Kim D. Y. Irimia D. (2018). Three‐dimensional models of the human brain development and diseases. Adv. Healthc. Mater. 7, 1700723. 10.1002/adhm.201700723 PMC5762251 28845922

[B20] Koehler R. C. Gebremedhin D. Harder D. R. (2006). Role of astrocytes in cerebrovascular regulation. J. Appl. Physiology 100, 307–317. 10.1152/japplphysiol.00938.2005 16357084 PMC1819408

[B21] Kolarova M. García-Sierra F. Bartos A. Ricny J. Ripova D. (2012). Structure and pathology of tau protein in alzheimer disease. Int. J. Alzheimers Dis. 2012, 1–13. 10.1155/2012/731526 22690349 PMC3368361

[B22] Liu B. Teschemacher A. G. Kasparov S. (2017). Neuroprotective potential of astroglia. J. Neurosci. Res. 95, 2126–2139. 10.1002/jnr.24140 28836687

[B23] Ma J. Ma C. Li J. Sun Y. Ye F. Liu K. (2020). Extracellular matrix proteins involved in alzheimer’s disease. Chem. – Eur. J. 26, 12101–12110. 10.1002/chem.202000782 32207199

[B24] Nakaji-Hirabayashi T. Kato K. Iwata H. (2013). *In vivo* study on the survival of neural stem cells transplanted into the rat brain with a collagen hydrogel that incorporates laminin-derived polypeptides. Bioconjug. Chem. 24, 1798–1804. 10.1021/bc400005m 23991904

[B25] Nasrollahi A. Yao Y. (2025). Laminins and the blood-brain barrier. Matrix Biol. 137, 33–41. 10.1016/j.matbio.2025.02.005 40032192 PMC12012582

[B26] Nho R. S. Hergert P. (2014). IPF fibroblasts are desensitized to type I collagen matrix-induced cell death by suppressing low autophagy via aberrant Akt/mTOR kinases. PLoS ONE 9, e94616. 10.1371/journal.pone.0094616 24728102 PMC3984186

[B27] Nimni M. E. (2018). Collagen: Volume I Biochemistry, 1st Edn ed Nimni M. E. , Boca Raton: CRC Press. 10.1201/9781351070799

[B28] O’Connor S. M. Stenger D. A. Shaffer K. M. Ma W. (2001). Survival and neurite outgrowth of rat cortical neurons in three-dimensional agarose and collagen gel matrices. Neurosci. Lett. 304, 189–193. 10.1016/S0304-3940(01)01769-4 11343834

[B29] Rabkin S. W. (2023). Collagen type IV as the link between arterial stiffness and dementia. Am. J. Transl. Res. 15, 5961–5971. 37969177 PMC10641358

[B30] Reale M. Costantini E. (2021). Cholinergic modulation of the immune system in neuroinflammatory diseases. Diseases 9, 29. 10.3390/diseases9020029 33921376 PMC8167596

[B31] Ricard-Blum S. (2011). The collagen family. Cold Spring Harb. Perspect. Biol. 3, a004978. 10.1101/cshperspect.a004978 21421911 PMC3003457

[B32] Schwarz D. Lipoldová M. Reinecke H. Sohrabi Y. (2022). Targeting inflammation with collagen. Clin. Transl. Med. 12, e831. 10.1002/ctm2.831 35604877 PMC9126324

[B33] Singh B. Fleury C. Jalalvand F. Riesbeck K. (2012). Human pathogens utilize host extracellular matrix proteins laminin and collagen for adhesion and invasion of the host. FEMS Microbiol. Rev. 36, 1122–1180. 10.1111/j.1574-6976.2012.00340.x 22537156

[B34] Steiner K. Humpel C. (2021). Microcontact printing of cholinergic neurons in organotypic brain slices. Front. Neurol. 12, 775621. 10.3389/fneur.2021.775621 34867765 PMC8636044

[B35] Steiner K. Humpel C. (2023). Long-term organotypic brain slices cultured on collagen-based microcontact prints: a perspective for a brain-on-a-chip. J. Neurosci. Methods 399, 109979. 10.1016/j.jneumeth.2023.109979 37783349

[B36] Summers L. Kangwantas K. Rodriguez-Grande B. Denes A. Penny J. Kielty C. (2013). Activation of brain endothelial cells by interleukin-1 is regulated by the extracellular matrix after acute brain injury. Mol. Cell. Neurosci. 57, 93–103. 10.1016/j.mcn.2013.10.007 24161715

[B37] Sweeney M. D. Kisler K. Montagne A. Toga A. W. Zlokovic B. V. (2018). The role of brain vasculature in neurodegenerative disorders. Nat. Neurosci. 21, 1318–1331. 10.1038/s41593-018-0234-x 30250261 PMC6198802

[B38] Tang M.-J. Hu J.-J. Lin H.-H. Chiu W.-T. Jiang S.-T. (1998). Collagen gel overlay induces apoptosis of polarized cells in cultures: disoriented cell death. Am. J. Physiol.-Cell Physiol. 275, C921–C931. 10.1152/ajpcell.1998.275.4.C921 9755045

[B39] Tarnowski B. I. Spinale F. G. Nicholson J. H. (1991). DAPI as a useful stain for nuclear quantitation. Biotech. Histochem. 66, 296–302. 10.3109/10520299109109990 1725854

[B40] Ucar B. Humpel C. (2018). Collagen for brain repair: therapeutic perspectives. Neural Regen. Res. 13, 595–598. 10.4103/1673-5374.230273 29722301 PMC5950659

[B41] Verkhratsky A. Nedergaard M. Hertz L. (2015). Why are astrocytes important? Neurochem. Res. 40, 389–401. 10.1007/s11064-014-1403-2 25113122

[B42] Walker L. C. (2020). Aβ plaques. Free Neuropathol, 31. 10.17879/FREENEUROPATHOLOGY-2020-3025 PMC7745791 33345256

[B43] Wareham L. K. Baratta R. O. Del Buono B. J. Schlumpf E. Calkins D. J. (2024). Collagen in the central nervous system: contributions to neurodegeneration and promise as a therapeutic target. Mol. Neurodegener. 19, 11. 10.1186/s13024-024-00704-0 38273335 PMC10809576

[B44] Weber A. Wasiliew P. Kracht M. (2010). Interleukin-1 (IL-1) pathway. Sci. Signal. 3, cm1. 10.1126/scisignal.3105cm1 20086235

[B45] Weis C. Marksteiner J. Humpel C. (2001). Nerve growth factor and glial cell line-derived neurotrophic factor restore the cholinergic neuronal phenotype in organotypic brain slices of the basal nucleus of meynert. Neuroscience 102, 129–138. 10.1016/S0306-4522(00)00452-8 11226676

[B46] Wilson S. L. Guilbert M. Sulé-Suso J. Torbet J. Jeannesson P. Sockalingum G. D. (2012). in The Effect of Collagen Ageing on Its Structure and Cellular Behaviour. Editors Tuchin V. V. Duncan D. D. Larin K. V. Leahy M. J. Wang R. K. (San Francisco, California, USA). 10.1117/12.908749 822210

[B47] Yilmaz S. N. Steiner K. Marksteiner J. Faserl K. Villunger M. Sarg B. (2024). From organotypic mouse brain slices to human Alzheimer’s plasma biomarkers: a focus on nerve fiber outgrowth. Biomolecules 14, 1326. 10.3390/biom14101326 39456259 PMC11506054

[B48] Yilmaz S. N. Steiner K. Marksteiner J. Faserl K. Sarg B. Humpel C. (2025). Novel plasma biomarkers for alzheimer’s disease: insights from organotypic brain slice and microcontact printing techniques. Front. Biosci. Landmark Ed. 30, 36257. 10.31083/FBL36257 40152394

[B49] Zapata-Acevedo J. F. García-Pérez V. Cabezas-Pérez R. Losada-Barragán M. Vargas-Sánchez K. González-Reyes R. E. (2022). Laminin as a biomarker of blood–brain barrier disruption under neuroinflammation: a systematic review. Int. J. Mol. Sci. 23, 6788. 10.3390/ijms23126788 35743229 PMC9224176

